# Floating Gate, Organic Field-Effect Transistor-Based Sensors towards Biomedical Applications Fabricated with Large-Area Processes over Flexible Substrates

**DOI:** 10.3390/s18030688

**Published:** 2018-02-26

**Authors:** Stefano Lai, Fabrizio Antonio Viola, Piero Cosseddu, Annalisa Bonfiglio

**Affiliations:** Department of Electrical and Electronic Engineering, University of Cagliari, Piazza d’Armi, 09123 Cagliari, Italy; fabrizio.viola@diee.unica.it (F.A.V.); piero.cosseddu@diee.unica.it (P.C.); annalisa@diee.unica.it (A.B.)

**Keywords:** inkjet printing, chemical vapor deposition, OTFTs, temperature sensing, pressure sensing

## Abstract

Organic Field-Effect Transistors (OFETs) are attracting a rising interest for the development of novel kinds of sensing platforms. In this paper, we report about a peculiar sensor device structure, namely Organic Charge-Modulated Field-Effect Transistor (OCMFET), capable of operating at low voltages and entirely fabricated with large-area techniques, i.e., inkjet printing and chemical vapor deposition, that can be easily upscaled to an industrial size. Device fabrication is described, and statistical characterization of the basic electronic parameters is reported. As an effective benchmark for the application of large-area fabricated OCMFET to the biomedical field, its combination with pyroelectric materials and compressible capacitors is discussed, in order to employ the proposed device as a temperature pressure sensor. The obtained sensors are capable to operate in conditions which are relevant in the biomedical field (temperature in the range of 18.5–50 °C, pressure in the range of 10^2^–10^3^ Pa) with reproducible and valuable performances, opening the way for the fabrication of low-cost, flexible sensing platforms.

## 1. Introduction

Following the evolution of technologies, end users of biomedical devices (technicians, physicians, and patients) expect that features like ease of use, low costs, non-invasiveness, and possibly unperceptiveness are completely exploited in novel products. If standard electronics can effectively fulfill some of these requirements, other aspects are far beyond the expected performance of silicon. As a valuable example, the development of wearable devices, which is thoroughly considered in the field of biomedical applications, requires large-area coverage, intrinsic flexibility, and lightness-in-weight that cannot be ensured by standard electronic processes and materials, in particular when low costs are targeted. In this view, the complementary development of novel classes of materials and technological processes where the above-mentioned features are addressed represents a fundamental research topic in modern bioengineering. Among the proposed technologies, plastic electronics is being thoroughly explored. Indeed, plastic substrates provide the required characteristics of flexibility, lightness-in-weight, and possibly optical transparency required for some novel applications. Among many different technological solutions, the employment of organic materials for the fabrication of electronic devices has gained a rising interest over the past years. Indeed, such materials are intrinsically soft and flexible and can be deposited over large areas using cost-effective technologies, such as printing processes. Moreover, their biocompatibility makes them a very interesting candidate for the fabrication of biosensing devices [1,2]. As a matter of fact, an increasing number of examples of organic device-based applications in the biomedical fields can be found in the literature: sensors of biological [3,4,5,6], chemical [7,8], and physical quantities [9,10,11,12,13] based on organic devices have been reported. In particular, Organic Thin-Film Transistors (OTFTs) have been explored. Their multiparametric nature and intrinsic signal amplification ability makes them interesting platforms for the development of different kinds of sensors, well beyond what is possible with two terminal devices.

Despite these interesting features, the actual diffusion of organic devices in real applications is still very limited. Indeed, to fill the gap between laboratory and real application environments, features like (i) fabrication with cost-effective techniques, (ii) low power consumption, (iii) process and performance reliability, and (iv) assessed working mechanisms, must be fulfilled at the same time. For instance, in the case of OTFT-based sensors, relatively high voltages are needed to obtain proper device functionality, thus reducing their actual portability and raising serious safety concerns for their application on-body and their integration with biochemical elements. Although several strategies for low voltage operation have been reported [14,15,16,17,18,19,20,21,22,23,24], only a few are exploited with large-area processes [25,26,27,28]. In these last cases, the application of OTFTs to sensing applications is not yet explored. Moreover, because in most cases the organic semiconductor is used as a sensing element, the actual performances are strongly reliant on the choice of the semiconductor, the reliability of its electrical properties as thin-film, and their deterioration. These features significantly vary among materials and fabrication methods. Therefore, the development of fabrication protocols that may be used at industrial size for the development of actual products is not trivial.

In this paper, we report on the development of a sensing platform based on floating-gate OTFTs for the actual application in the field of biomedical signal sensing. The device, namely, Organic Charge-Modulated Field-Effect Transistor (OCMFET), has been already employed as a valuable platform for different kinds of sensing applications, including the detection of biochemical reactions [29,30,31,32] and of physical quantities such as temperature [33,34] and pressure [35,36,37]. Here, we demonstrate that OCMFET-based devices for biosensing applications can be produced by means of large-area processes with significant performance reliability. In particular, inkjet printing and Chemical Vapor Deposition (CVD) are employed for the development of the entire device structure. Electrical characterization of the fabricated OCMFET structures is provided, showing the overall reliability and reproducibility of the fabrication process. Temperature and pressure sensing are considered as valuable benchmarks for the platform, and a complete characterization of the device performances is reported, focusing on ranges of stimuli which are significant for biomedical applications.

## 2. Materials and Methods

### 2.1. Fabrication of OCMFETs

**Figure 1:**
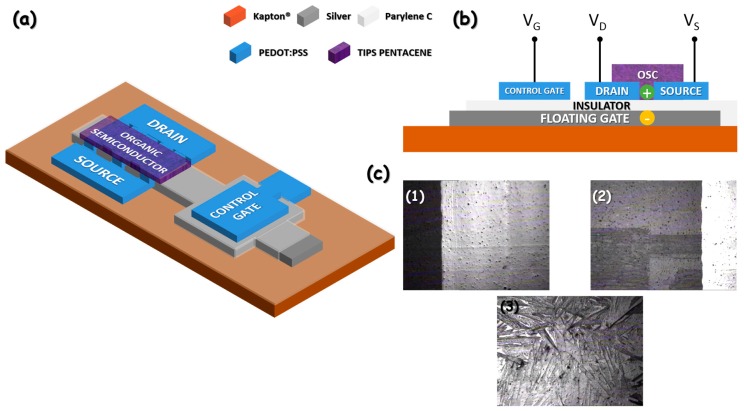
(**a**,**b**): structure of the Organic Charge-Modulated Field-Effect Transistor (OCMFET); (**c**) pictures of the printed layers acquired with the DMP2831 fiducial camera: (**1**) floating gate; (**2**) source/drain interdigitated pattern; (**3**) 6,13-Bis(triisopropylsilylethynyl)pentacene (TIPS pentacene).

The OCMFET structure is shown in Figure 1. Such a device has been obtained as a modification of the OFET structure previously presented in Lai et al. [27], capable to be operated at low voltages, with significant reliability of device performances and electrical and mechanical robustness (bending at a radius of 7 mm for 1000 times without any loss of functionality). Devices are fabricated over 125-µm-thick polyimide substrates (Kapton^®^, Goodfellow, Huntingdon, UK). Substrates were cleaned by a subsequent rinse of acetone, isopropyl alcohol, and deionized water. Oxygen plasma activation was performed using a Gambetti–Tucano plasma system, under a pressure of 0.2 mbar with a RF power of 60 W applied for 30 s. Plasma activation was used to increase the wettability of the Kapton^®^’s surfaces. The OCMFET floating gate was obtained by inkjet printing a silver-nanoparticles ink (Cabot CCI-330, Cabot, Boston, MA, USA) using a Dimatix Inkjet Printer DMP2831 (Fujifilm Dimatix, Santa Clara, CA, USA) equipped with 16-nozzle cartridges with a single drop volume of 1 pL. Inkjet printing was performed using a single nozzle, with a drop spacing of 25 µm, a firing voltage of 25 V, and a tickle frequency of 5 kHz. Silver ink sintering was performed in an oven at a temperature of 220 °C for 10 s. As previously reported in Lai et al. [27], the combination of surface activation, minimum ink employment, and relatively high sintering temperature obtains uniform, continuous, and conductive silver patterns with a thickness comparable with those obtained in physical deposition processes (in the range of 200 nm). This feature is fundamental for the fabrication of low-voltage OFETs. Indeed, in order to achieve low operating voltages, it is necessary to decrease the gate insulator thickness (i.e., increase the capacitance per unit area of the gate dielectric), but effective and reliable insulation over large areas of relatively thick and poorly uniform printed patterns is not trivial. The sheet resistance of the floating gate is relatively low (1.8 Ω/sq), but it is important to underline that in the proposed structure no current flows in the floating gate, so its actual resistivity does not play a role in device functionality. After the printing and sintering of the floating gate, CVD was used to deposit a thin layer (200 nm) of Parylene C using a PDS2010 unit (Specialty Coating Systems, Woking, UK). CVD is an industrial technique that can be easily integrated with inkjet printing and upscaled to roll-to-roll processes, allowing for the deposition of uniform, thin insulator layers over a large area with significant reliability. The surface of the deposited Parylene C layer is pinhole-free, inert, and robust to chemical treatment, thus being ideal for the subsequent fabrication steps needed for completing the OCMFET structure. The final capacitance per area unit obtained was 9 nF/cm^2^. Source, drain, and control capacitor were inkjet-printed using a poly(3,4-ethylenedioxythiophene):polystyrene sulfonate (PEDOT:PSS)-based commercial ink (PJET HC, Heraeus, Hanau, Germany). PEDOT:PSS was chosen for source and drain patterning, as its work function (of about 5 eV) is well suited for hole injection in p-type organic semiconductors. A cartridge with 16 nozzles and a single drop volume of 1 pL was also employed in this case. A drop spacing of 20 µm was employed, while firing voltage, number of nozzles, tickle frequency, and number of printed layers were varied according to the defined pattern. During the printing, the temperature of the platen was set to 60 °C. In the transistor area, an average aspect ratio of 180 was obtained (W/L = 18 mm/100 µm, W being the channel width and L the channel length) using an interdigitated pattern. A resolution in the range of 50 µm was obtained, with a sheet resistance of about 7 kΩ/sq. As the aspect ratio of each electrode in the interdigitated pattern is 10 (2 mm/200 µm), and 10 electrodes are used, a total resistance of 7 kΩ is obtained, thus being negligible if compared to the injection resistance of OFETs (in the range of several hundreds of kΩ [38]). A control capacitor of 3.5 × 3.5 mm^2^ was deposited. After the printing, the PEDOT:PSS was dried in oven at 120 °C for 15 min to improve insolubility to the organic solvents used for the subsequent fabrication steps. After that, Ethylene Glycol (EG, Sigma-Aldrich) was inkjet-printed to the PEDOT:PSS surface using the DMP2831, a 16-nozzle cartridge with a 10 pL volume for single drop, employing two nozzles, a firing voltage of 40 V, a drop spacing of 50 µm, and a tickle frequency of 7 kHz. Post-processing of PEDOT:PSS with EG is a common procedure to improve its conductivity and reduce its solubility in organic solvents [39]. EG was used without further dilutions, as its viscosity (16.9 cP at room temperature) and surface tension (48 dynes/cm at room temperature) are compatible with the printer specifications. EG was dried over the DMP2831 platen at 60 °C for 30 min. 6,13-Bis(triisopropylsilylethynyl)pentacene (TIPS pentacene, Sigma-Aldrich, Saint Luis, MO, USA) was chosen as the p-type, organic semiconductor. A solution containing 1 wt% of TIPS pentacene in anisole (Sigma-Aldrich) was prepared and used as ink with a 10 pL cartridge. TIPS pentacene was inkjet-printed using a drop spacing of 30 µm, 5 nozzles, a firing voltage of 40 V, and a tickle frequency of 7 kHz. The final ink has a viscosity of about 1 cP at room temperature and a surface tension of about 30 dynes/cm at room temperature. The deposited pattern was let to dry at room temperature over the platen of the DMP2831.

### 2.2. Temperature-Sensitive Capacitors

Polyvinylidene fluoride (PVDF) was used as the pyroelectric material. A 28-µm-thick PVDF film, already poled (Measurement Specialties Inc.-MEAS, Hampton, VA, USA) was sandwiched between two metal layers in order to form a capacitor (area = 5 × 5 mm^2^, C_INS_ = 110 pF/cm^2^) and connected by a metal wire with the floating gate of the transistor. The two electrodes were made of silver and patterned by inkjet printing, using a 16-nozzle cartridge with 10 pL volume for single drop, a drop spacing of 20 μm, a firing voltage 30 V, a tickle frequency 5 kHz, and then dried in an oven at 60 °C, well below the Curie temperature of the PVDF [40].

### 2.3. Pressure-Sensitive Capacitors

A pressure-sensitive capacitor made of Polydimethylsiloxane (PDMS, Sylgard 184, Dow Corning, Midland, MI, USA) was fabricated and connected to the floating gate of the OCMFET with a metal wire. The bottom plate of the capacitor was fabricated using silver ink, according to the procedure described for the floating gate; a 200-nm-thick Parylene C layer was deposited on the bottom plate to enhance PDMS adhesion. PDMS was prepared by mixing the elastomer and the curing agent (10:1 in weight); the mix was degassed in a vacuum and then deposited on the bottom plate of the capacitor. A 40-µm-thick layer was obtained by spin-coating the PDMS for 2 min at 6000 rpm and then drying it in an oven at 100 °C for an hour. A top layer of Parylene C (200 nm) was deposited onto the PDMS layer to promote the adhesion of the top plate, obtained by thermal evaporation of silver at high-vacuum (10^−4^ Torr) through a shadow mask. The final area of the capacitor is 5 × 5 mm^2^, with a capacitance value of 90 pF (while not compressed). This capacitance is mainly related to the PDMS layer being thicker than the two Parylene C layers employed as adhesion layers, and the relative dielectric constant of the two materials is similar (ε_PARYLENE_ = 3.15, ε_PDMS_ = 2.7, values considered at 60 Hz).

### 2.4. Device Characterization

OCMFETs have been electrically characterized using a Keithley SourceMeter^®^ 2614, controlled with custom MATLAB^®^ scripts. For temperature-sensing tests, the PVDF capacitor was placed on a Peltier cell (Farnell, Leeds, UK); an optical pyrometer (PyroCouple PC21MT-1, CALEX Electronics Limited, Leighton Buzzard, UK) allowed temperature evaluation on the Peltier cell, with parallel data acquisition for sensor calibration. Compressible capacitors were characterized using a 1 HP 4182 Impedance Analyzer (Agilent, Santa Clara, CA, USA).

## 3. Results and Discussion

### 3.1. OCMFET Working Principle

As previously reported [41], the OCMFET is a modified extended-gate structure, where the gate bias is applied through a control electrode capacitively coupled with the floating gate. A part of the floating gate, not covered by the insulator, is a sensing area that allows employing the OCMFET as a charge sensor. The floating gate voltage is given by

(1) $$V_{\mathrm{FG}}=\frac{C_{\mathrm{CG}}}{C_{\mathrm{CG}}+C_{\mathrm{DF}}+C_{\mathrm{SF}}}V_{\mathrm{GS}}+\frac{C_{\mathrm{DF}}}{C_{\mathrm{CG}}+C_{\mathrm{DF}}+C_{\mathrm{SF}}}V_{\mathrm{DS}}+\frac{Q_{0}+Q_{i}\left(Q_{S}\right)}{C_{\mathrm{CG}}+C_{\mathrm{DF}}+C_{\mathrm{SF}}},$$

where C_CG_, C_DF_, and C_SF_ are the capacitance between control gate, source and drain, and the floating gate, respectively, V_GS_ and V_DS_ are the voltage drop between control gate/drain and source, respectively, Q_0_ the intrinsic charge of the floating gate, and Q_i_(Q_S_) is the induced charge in the floating gate by a charge Q_S_ in the near proximity of the sensing area. This voltage is the actual voltage of the OFET structure: for instance, the current I_DS_ flowing in the transistor in the saturation regime (|V_DS_| > |V_GS_ − V_TH_|) can be thus written as

(2) $$I_{\mathrm{DS}}=\frac{1}{2}\mu C_{\mathrm{INS}}\frac{W}{L}{\left(V_{\mathrm{FG}}-V_{\mathrm{TH}}\right)}^{2},$$

where µ is the charge carrier mobility, C_INS_ is the capacitance per unit area of the gate insulator, W and L are channel width and length, respectively, and V_TH_ is the threshold voltage of the transistor. If C_CG_ is sufficiently larger than C_DF_ and C_SF_, and a perfect induction is supposed (Q_i_(Q_S_) = −Q_S_), the latter equation can be written as

(3) $$I_{\mathrm{DS}}=\frac{1}{2}\mu C_{\mathrm{INS}}\frac{W}{L}{\left[V_{\mathrm{CG}}-\left(V_{\mathrm{TH}}-\frac{Q_{0}-Q_{S}}{C_{\mathrm{CG}}}\right)\right]}^{2}.$$

When Q_S_ changes, a variation in the output current is therefore induced; this variation can be modelled as a variation in the threshold voltage ΔV_TH_

(4) $$\Delta V_{\mathrm{TH}}=-\frac{\Delta Q_{S}}{C_{\mathrm{CG}}},$$

being ΔQ_S_ the detected variation in Q_S_.

### 3.2. Electrical Characterization

**Figure 2:**
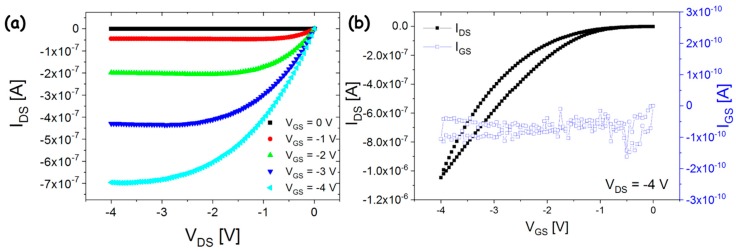
Typical output (**a**) and transfer (**b**) characteristic curves of fabricated OCMFETs.

In Figure 2, typical output and transfer characteristic curves of the OCMFET are reported. Twenty devices have been successfully fabricated and tested. A short-circuit between the control gate and the floating gate was observed in only one device. A total process yield of 95% was thus obtained, which is particularly relevant for fabrication with academic laboratory facilities. Devices have an average threshold voltage of 0.8 ± 0.1 V, thus being capable to operate at low voltages (|V_DS_, V_GS_| below 5 V). Average charge carrier mobility in the saturation regime, as extracted by the square root of the transfer characteristic curve [42], is (7 ± 2) × 10^−2^ cm^2^ V^−1^ s^−1^; leakage current is in the range of a few tens of pA. This last aspect is particularly important in OCMFET structures: a too high leakage current flowing between the control gate and the source would indicate that the insulation of the floating gate is scarce, i.e., the charge in the floating gate may vary during the measurement, thus affecting the reliability of the measurement. From the transfer characteristic curve, a limited, though not negligible, hysteresis can be also noticed, thus proving the rather good characteristics of injection from source/drain contacts and the organic semiconductor, and of the semiconductor/insulator interface.

### 3.3. Characterization of OCMFETs as Temperature Sensors

**Figure 3:**
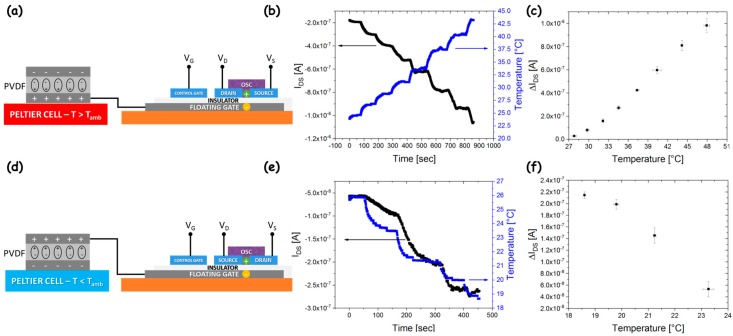
(**a**) connection of the OCMFET with the PVDF capacitor for a temperature-sensing test with T > T_amb_; (**b**) example of current variation (black curve) recorded in real time for increasing temperatures, monitored by an optical pyrometer (blue curve); (**c**) calibration curve as absolute current variation vs. temperature (each point averaged over five measurements); (**d**) connection of the OCMFET with the PVDF capacitor for a temperature-sensing test with T < T_amb_; (**e**) example of current variation (black curve) recorded in real time for decreasing temperatures, monitored by an optical pyrometer (blue curve); (**f**) calibration curve as absolute current variation vs. temperature (each point averaged over five measurements).

In order to exploit the working principle described in Section 3.1 for temperature sensing, fabricated OCMFET structures where connected with PVDF capacitors. The floating gate was connected with a plate of the PVDF capacitor by means of a metal wire, while the other plate was left floating during the experiment (Figure 3a). The working principle of the temperature sensors is very simple. As already reported in Cosseddu et al. [33], when a temperature variation is induced on the sensing area, a charge separation in the PVDF film is induced, thus leading to a OCMFET threshold voltage variation, which, in turn, leads to a variation of the output current.

Since PVDF has a non-zero polarization vector, the choice of the plate connected to the floating gate changes the way the OCMFET will respond to temperature variations provided by a Peltier cell; the entity of temperature variations was monitored in real time using an optical pyrometer. Figure 3b shows the variation of the output current recorded in real time while the temperature of the Peltier cell was progressively increased with respect to the room temperature (T_amb_ = 27 °C). It is possible to observe that the current increases, with a dynamic similar to the one of the cell, as acquired by the optical pyrometer. According to Equation (4), the current increase is related to a reduction of the threshold voltage, which is due to an increase of the holes concentration in the active channel; therefore, a positive charge is induced in the plate of the PVDF capacitor connected to the floating gate. In Figure 3c, the calibration curve showing the absolute current variation as a function of the temperature is reported: the data are an average over five different measurements. A good linearity of the response and a significant reproducibility were obtained. On a set of five devices tested, an average sensitivity of about 50 ± 5 nA/°C was obtained. When temperatures lower than T_amb_ are considered, a current reduction is obtained: an example of the heating–cooling cycle is shown in Figure S1 of the Supplementary Material. Even if proper device functionality can be observed when the transistor is switched off by the temperature decrease, a reduction of signal-to-noise ratio in the sensor response is obtained in these conditions. In order to transduce reduction of the temperature with respect to T_amb_ with current increase (i.e., with an increase of the signal-to-noise ratio), the floating gate can be connected to the opposite plate of the PVDF capacitor (Figure 3d). In this way, the temperature decrease induces a positive charge on the top plate, and holes can be attracted in the transistor channel, thus determining a current increase. Figure 3e,f show the results of characterization for T < T_amb_: also in this case, a dynamics comparable to those of the optical pyrometer was obtained, and the response result was quite reproducible. Different from the previous experiment, an evident saturation of the response can be observed for the lower value of temperature considered (T = 18.5 °C): this reduced linearity range can be ascribed to the charge carrier density induced by the top plate in the transistor channel at T = T_amb_ that determines a shift of the transistor working point close to the saturation of the response. In conclusion, an overall linear range of the current variation between 18.5–50 °C, with reproducible response over multiple experiments, was obtained, proving the reliability of the device performance in conditions that are relevant for several biomedical applications, such as fabrication of artificial skin and temperature monitoring of biochemical experiments and cell cultures.

### 3.4. OCMFETs as Pressure-Sensitive Ofets

Pressure detection and transduction is of great interest in biomedical applications: it is fundamental for reproducing the sense of touch in artificial skin or for the transduction of blood pressure in health monitoring systems. The OCMFET structure can be easily converted into a Pressure-Modulated OFET (PMOFET), by substituting the sensing area with a compressible capacitor. In this way, Equation (1) can be rewritten as

(5) $$\begin{array}{l} V_{\mathrm{FG}}=\frac{C_{\mathrm{CG}}}{C_{\mathrm{CG}}+C_{\mathrm{DF}}+C_{\mathrm{SF}}+C_{P}}V_{\mathrm{GS}}+\frac{C_{\mathrm{DF}}}{C_{\mathrm{CG}}+C_{\mathrm{DF}}+C_{\mathrm{SF}}+C_{P}}V_{\mathrm{DS}}+\frac{C_{P}}{C_{\mathrm{CG}}+C_{\mathrm{DF}}+C_{\mathrm{SF}}+C_{P}}V_{\mathrm{PS}}+\\ +\frac{Q_{0}}{C_{\mathrm{CG}}+C_{\mathrm{DF}}+C_{\mathrm{SF}}+C_{P}} \end{array},$$

where C_P_ is the capacitance of the compressible capacitor and V_PS_ the voltage applied on it (with respect to the source of the transistor). If Q_0_ is negligible, C_GS_ > C_P_ >> C_DF_, C_SF_, and if V_PS_ is opportunely chosen, the modulation of the floating gate voltage can be written as

(6) $$\Delta V_{\mathrm{TH}}\approx \frac{\Delta C_{P}}{C_{\mathrm{CG}}}V_{\mathrm{PS}},$$

where ΔC_P_ is the variation of the compressible capacitance upon the application of a pressure. Therefore, the floating gate structure is converted from a charge sensor to a device sensitive to the application of a mechanical deformation over the compressible capacitor. This principle has been previously reported in Lai et al. for a structure fabricated with small area, laboratory-scale techniques, showing the possibility of transducing the capacitive variation related to pressure stimuli with current variations that are easily acquired and conditioned by acquisition electronics. Moreover, for a given stimulus, an amplification effect due to the transistor structure was observed, i.e., the relative current variation was larger than the bare capacitive variation that generates the current modulation itself.

**Figure 4:**
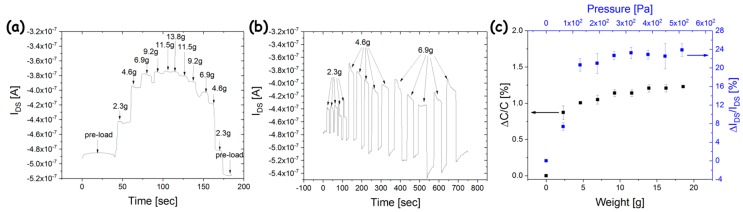
(**a**) PMOFET output current recorded in real time while an increasing weight was applied to the PDMS compressible capacitor; (**b**) the same experiment was repeated by subsequently applying and removing different weights for five times each; (**c**) capacitive variation (black squares, left *Y* axis) and corresponding current variation in the PMOFET (blue squares, right *Y* axis) as a function of increasing weight; corresponding pressure values are reported on the top *X* axis.

To obtain a PMOFET, the fabricated OCMFETs were connected with a metal wire to a compressible capacitor made of PDMS. As reported in the Materials and Methods Section, the PDMS capacitor was fabricated by means of spin coating, and, interestingly enough, the printability of PDMS has been demonstrated through large nozzle printing facilities [43]. The device has been tested for pressure sensing in the case of small pressure applied (in the range of 10^2^–10^3^ Pa), which is particularly interesting for biomedical applications, such as sense-of-touch mimic and evaluation of biosignals related to pressure variations. Pressure was applied to the capacitor using copper weights, each with a weight of 2.3 g and an area of 100 mm^2^, thus completely covering the area of the compressible capacitor. Multiple weights were used during the measurements. A plastic sheet was used to prevent direct contact between the weights and the top plate of the capacitor and to set the preload of the structure. In Figure 4a, the current variation in the transistor structure recorded in real time during the application of an increasing pressure is shown. For weight values below 4.6 g (corresponding to a pressure of about 130 Pa), the current variation is almost linear with a sensitivity of about 25 nA/g, then a sublinear response can be noticed as the limit of compression for the PDMS capacitor is approached. When such a limit is reached (weight ≥ 9.2 g, i.e., pressure ≥ 190 Pa), no significant current variations are observed, with only small transient variations recorded related to the capacitive currents induced when the capacitance changes. After a maximum weight of 13.8 g (corresponding to a pressure of 380 Pa) was applied, the weights were removed one by one. As a result, the device current progressively decreased, coming back to the previous current values. In the device recovery, an evident capacitive transient can be noticed, changing the current baseline of the sensor: this quite slow transient is related to the viscoelastic behavior and the mechanical inertia of the PDMS during relaxation. The time constant of the capacitive response at full load was evaluated to be in the range of 300 s. It is noteworthy that such an effect is related to the relatively long experiment, during which the system remained subjected to a continuous pressure for a prolonged time, which is quite an unlikely condition in most applications. As a matter of fact, when each stimulus was applied for a reduced amount of time (Figure 4b), the current baseline was well recovered in a reduced amount of time; only a slight reduction (in the range of 40 nA), related to the transistor bias stress, can be noticed at the end of the series of measurements. In this experiment, different pressure values were tested (below the saturation value): each weight was applied and removed five times in order to examine the reproducibility of the current response, which resulted in effective results for each series of stimuli. The same experiments were carried out for all the different weights considered, in order to evaluate the average current variation. In Figure 4c, the percentage variation of the output current is reported as a function of the applied pressure (blue squares); in the same plot, the percentage capacitive variation (black squares), recorded by means of an impedance analyzer on the capacitor subject to the same pressure values employed during the PMOFET characterization, is reported. In this case, an evident amplification in the stimuli transduction is obtained; the correct choice of capacitor area and applied voltage, and the correct design of the transistor structure obtained an average amplification of about 24 ± 2% at full pressure scale (average amplification evaluated on a set of five devices), well beyond previously reported findings [35]. An optimization of the device performances in terms of linearity and extent of the response are related to a final optimization of the characteristic of the capacitive element (e.g., PDMS thickness and capacitor area), which are outside the scope of the present work. Nonetheless, the obtained results demonstrate that printed OCMFET can be used as a straightforward platform for capacitive pressure sensors transduction with intrinsic amplification.

## 4. Conclusions

In this paper, we demonstrate the possibility of reliably fabricating floating-gate OFET structures by means of large-area techniques, such as inkjet printing and chemical vapor deposition, to be employed as flexible sensing platform in biomedical applications. A complete characterization of the devices was provided: over a few tens of fabricated structures, a process yield of 95% was obtained, with a good reproducibility of the device performances in terms of threshold voltage and charge carrier mobility. The fabricated devices were capable to operate at low voltages, which is a fundamental requirement for correct and safe employment in biomedical applications and for an actual portability of the devices. As relevant benchmarks for biomedical applications, the fabricated devices were tested as temperature and pressure sensors by coupling them with pyroelectric elements and compressible capacitors. As a temperature sensor, the devices demonstrated good and reproducible responses and a sensitivity of 50 ± 5 nA/°C in the range 18.5–50 °C, which is relevant in several biomedical applications. As a pressure sensor, the capability of the devices to operate in the pressure range 10^2^ ÷ 10^3^ Pa with reproducible performances was demonstrated. The amplitude of the operating range is obviously given by the mechanical characteristics of the compressible capacitor, which can be tuned according to the addressed applications. In any case, the obtained PMOFET was able to transduce the pressure event into a current variation, which is easier to be further conditioned by readout electronics than capacitive variation, and provides a significant amplification with respect to the pure capacitive variation. Although sensing performances still need to be optimized in order to make such devices suitable for application in real-life scenarios, these benchmarks provide a proof-of-concept demonstration about the actual feasibility of the proposed technology to the development of sensing platforms for biomedical applications with cost-effective techniques that can be easily upscaled to an industrial size. This may include the employment of larger inkjet printing systems that can be found at production levels with larger printing area (several m^2^) and more sophisticated printheads (with hundreds of nozzles), or other printing facilities, such as roll-to-roll printers. CVD units with larger chamber volume are also available. Further activities related to the exploitation of the OCMFET approach in large-area technologies to other kinds of biomedical devices, such as biochemical sensors, are currently ongoing.

## Supplementary Materials

The following are available online at http://www.mdpi.com/1424-8220/18/3/688/s1, Figure S1: example of heating/cooling cycle.

## References

[1] Søndergaard R.R., Hösel M., Krebs F.C. (2013). Roll-to-Roll Fabrication of Large Area Functional Organic Materials. J. Polym. Sci. B Polym. Phys..

[2] Mattana G., Loi A., Woytasik M., Barbaro M., Noël V., Piro B. (2017). Inkjet-Printing: A New Fabrication Technology for Organic Transistors. Adv. Mater. Technol..

[3] Zhang Q., Subramanian V. (2007). DNA hybridization detection with organic thin film transistors: Toward fast and disposable DNA microarray chips. Biosens. Bioelectron..

[4] Khan H.U., Roberts M.E., Johnson O., Förch R., Knoll W., Bao Z. (2010). In Situ, Label-Free DNA Detection Using Organic Transistor Sensors. Adv. Mat..

[5] Kergoat L., Piro B., Berggren M., Pham M.-C., Yassar A., Horowitz G. (2012). DNA detection with a water-gated organic field-effect transistor. Org. Electron..

[6] Medina-Sánchez M., Martínez-Domingo C., Ramon E., Merkoçi A. (2014). An Inkjet-Printed Field-Effect Transistor for Label-Free Biosensing. Adv. Funct. Mater..

[7] Bartic C., Palan B., Campitelli A., Borghs G. (2002). Monitoring pH with organic-based field-effect transistors. Sens. Actuator B Chem..

[8] Bartic C., Campitelli A., Borghs S. (2003). Field-effect detection of chemical species with hybrid organic/inorganic transistors. Appl. Phys. Lett..

[9] Zirkl M., Haase A., Fian A., Schön H., Sommer C., Jakopic G., Leising G., Stadlober B., Graz I., Gaar N., Schwödiauer R., Bauer-Gogonea S., Bauer S. (2007). Low-Voltage Organic Thin-Film Transistors with High-*k* Nanocomposite Gate Dielectrics for Flexible Electronics and Optothermal Sensors. Adv. Mater..

[10] Jung S., Ji T., Varadan V.K. (2007). Temperature sensor using thermal transport properties in the subthreshold regime of an organic thin film transistor. Appl. Phys. Lett..

[11] Jiseok K., Ng T.N., Kim W.S. (2012). Highly sensitive tactile sensors integrated with organic transistors. Appl. Phys. Lett..

[12] Schwartz G., Tee B.C.K., Mei J., Appleton A.L., Kim D.H., Wang H., Bao Z. (2013). Flexible polymer transistors with high pressure sensitivity for application in electronic skin and health monitoring. Nat. Commun..

[13] Someya T., Kato Y., Sekitani T., Iba S., Noguchi Y., Murase Y., Kawaguchi H., Sakurai T. (2005). Conformable, flexible, large-area networks of pressure and thermal sensors with organic transistor active matrixes. Proc. Natl. Acad. Sci. USA.

[14] Klauk H., Zschieschang U., Halik M. (2007). Low-voltage organic thin-film transistors with large transconductance. J. Appl. Phys..

[15] Zschieschang U., Ante F., Yamamoto T., Takimiya K., Kuwabara H., Ikeda M., Sekitani T., Someya T., Kern K., Klauk H. (2010). Flexible Low-Voltage Organic Transistors and Circuits Based on a High-Mobility Organic Semiconductor with Good Air Stability. Adv. Mater..

[16] Cosseddu P., Lai S., Barbaro M., Bonfiglio A. (2012). Ultra-low voltage, organic thin film transistors fabricated on plastic substrates by a highly reproducible process. Appl. Phys. Lett..

[17] Machado W.S., Hummelgen I.A. (2012). Low-Voltage Poly(3-Hexylthiophene)/Poly(Vinyl Alcohol) Field-Effect Transistor and Inverter. IEEE Trans. Electron Devices.

[18] Kaltenbrunner M., Sekitani T., Reeder J., Yokota T., Kuribara K., Tokuhara T., Drack M., Schwödiauer R., Graz I., Bauer-Gogonea S., Bauer S., Someya T. (2013). An ultra-lightweight design for imperceptible plastic electronics. Nature.

[19] Han C.Y., Tang W.M., Leung C.H., Che C.M., Lai P.T. (2013). High-Performance Pentacene Thin-Film Transistor with High-κ HfLaON as Gate Dielectric. IEEE Electron. Device Lett..

[20] Zhao X., Wang S., Li A., Ouyang J., Xia G., Zhou J. (2014). Universal solution-processed high-κ amorphous oxide dielectrics for high-performance organic thin-film transistors. RSC Adv..

[21] Tetzner K., Schroeder K.A., Bock K. (2014). Photonic curing of sol–gel derived HfO_2_ dielectrics for organic field-effect transistors. Ceram. Int..

[22] Tang W.M., Aboudi U., Provine J., Howe T., Wong H.-S.P. (2014). Improved performance of bottom-contact organic thin-film transistor using Al doped HfO_2_ gate dielectric. IEEE Trans. Electron Devices.

[23] Sandberg H.G.O., Bäcklund T.G., Österbacka R., Stubb H. (2004). High-Performance All-Polymer Transistor Utilizing a Hygroscopic Insulator. Adv. Mater..

[24] Tobjörk D., Kaihovirta N.J., Mäkelä T., Pettersson F., Österbacka R. (2008). All-printed low-voltage organic transistors. Org. Electron..

[25] Grau G., Subramanian V. (2016). Fully High-Speed Gravure Printed, Low-Variability, High-Performance Organic Polymer Transistors with Sub-5 V Operation. Adv. Electron. Mater..

[26] Feng L., Jiang C., Ma H., Guo X., Nathan A. (2016). All ink-jet printed low-voltage organic field-effect transistors on flexible substrate. Org. Electron..

[27] Lai S., Cosseddu P., Zucca A., Loi A., Bonfiglio A. (2017). Combining inkjet printing and chemical vapor deposition for fabricating low voltage, organic field-effect transistors on flexible substrates. Thin Solid Films.

[28] Conti S., Lai S., Cosseddu P., Bonfiglio A. (2017). An Inkjet-Printed, Ultralow Voltage, Flexible Organic Field Effect Transistor. Adv. Mater. Technol..

[29] Demelas M., Lai S., Casula G., Scavetta E., Barbaro M., Bonfiglio A. (2012). An organic, charge-modulated field effect transistor for DNA detection. Sens. Actuator B Chem..

[30] Lai S., Demelas M., Casula G., Cosseddu P., Barbaro M., Bonfiglio A. (2013). Ultralow Voltage, OTFT-Based Sensor for Label-Free DNA Detection. Adv. Mater..

[31] Lai S., Barbaro M., Bonfiglio A. (2016). Tailoring the sensing performances of an OFET-based biosensor. Sens. Actuator B Chem..

[32] Spanu A., Viola F.A., Lai S., Cosseddu P., Ricci P.C., Bonfiglio A. (2017). A reference-less pH sensor based on an organic field effect transistor with tunable sensitivity. Org. Electron..

[33] Cosseddu P., Viola F.A., Lai S., Raffo L., Bonfiglio A. (2014). A Temperature Transducer Based on a Low Voltage Organic Thin-Film Transistor Detecting Pyroelectric Effect. IEEE Electron Device Lett..

[34] Viola F., Cosseddu P., Lai S., Spanu A., Bonfiglio A. Flexible Temperature Sensors Based on Charge Modulated Organic Thin Film. Proceedings of the 11th Conference on Ph.D. Research in Microelectronics and Electronics (PRIME).

[35] Lai S., Cosseddu P., Bonfiglio A., Barbaro M. (2013). Ultralow Voltage Pressure Sensors Based on Organic FETs and Compressible Capacitors. IEEE Electron Device Lett..

[36] Spanu A., Pinna L., Viola F.A., Seminara L., Valle M., Bonfiglio A., Cosseddu P. (2016). A high-sensitivity tactile sensor based on piezoelectric polymer PVDF coupled to an ultra-low voltage organic transistor. Org. Electron..

[37] Cosseddu P., Viola F., Lai S., Raffo L., Seminara L., Pinna L., Valle M., Dahiya R., Bonfiglio A. (2014). Tactile sensors with integrated piezoelectric polymer and low voltage organic thin-film transistors. IEEE Sensors.

[38] Lai S., Cosseddu P., Bonfiglio A. (2017). A method for direct contact resistance evaluation in low voltage coplanar organic field-effect transistors. App. Phys. Lett..

[39] Ouyang J., Xu Q., Chu C.-W., Yang Y., Li G., Shinar J. (2004). On the mechanism of conductivity enhancement in poly (3, 4-ethylenedioxythiophene): Poly (styrene sulfonate) film through solvent treatment. Polymer.

[40] Lovinger A.J., Davis D.D., Cais R.E., Kometani J.M. (1986). On the Curie temperature of poly (vinylidene fluoride). Macromolecules.

[41] Demelas M., Lai S., Spanu A., Martinoia S., Cosseddu P., Barbaro M., Bonfiglio A. (2013). Charge sensing by organic charge-modulated field effect transistors: Application to the detection of bio-related effects. J. Mater. Chem. B.

[42] Horowitz G., Lang P., Mottaghi M., Aubin H. (2004). Extracting Parameters from the Current-Voltage Characteristics of Organic Field-Effect Transistors. Adv. Funct. Mater..

[43] Peng Y., Xiao S., Yang J., Lin J., Yuan W., Gu W., Wu X., Cui Z. (2017). The elastic microstructures of inkjet printed polydimethylsiloxane as the patterned dielectric layer for pressure sensors. Appl. Phys. Lett..

