# Supplementary material for: Floating Gate, Organic Field-Effect Transistor-Based Sensors towards Biomedical Applications Fabricated with Large-Area Processes over Flexible Substrates
Source: Sensors (Basel). 2018 Feb 26;18(3):688. doi: 10.3390/s18030688 (PMC5876878; doi:10.3390/s18030688)
Supplement: Supplementary file 1 [file sensors-18-00688-s001.pdf]

Supplementary Materials

Floating Gate, Organic Field-Effect Transistor-Based Sensors towards Biomedical Applications Fabricated with Large-Area Processes over Flexible Substrates

Stefano Lai *, Fabrizio Antonio Viola , Piero Cosseddu and Annalisa Bonfiglio

Department of Electrical and Electronic Engineering, University of Cagliari, Piazza d'Armi, 09123 Cagliari, Italy; fabrizio.viola@diee.unica.it (F.A.V.); piero.cosseddu@diee.unica.it (P.C.); annalisa@diee.unica.it (A.B.)

* Correspondence: stefano.lai@diee.unica.it; Tel.: +39-070-675-5769

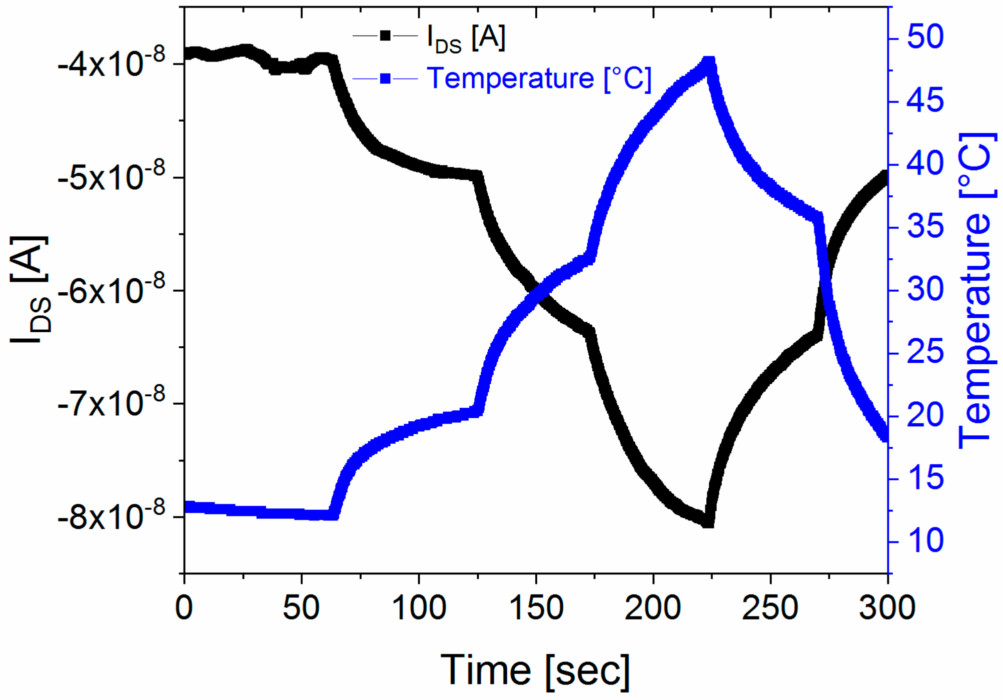

Figure S1: Example of heating/cooling cycle.
